# Compete or Cooperate with ‘Dr. Google’? Small Animal Veterinarians’ Attitudes towards Clients’ Use of Internet Resources—A Comparative Study across Austria, Denmark and the UK

**DOI:** 10.3390/ani12162117

**Published:** 2022-08-18

**Authors:** Svenja Springer, Herwig Grimm, Peter Sandøe, Thomas Bøker Lund, Annemarie T. Kristensen, Sandra A. Corr

**Affiliations:** 1 Unit of Ethics and Human-Animal Studies, Messerli Research Institute, University of Veterinary Medicine, Vienna, Medical University of Vienna, University of Vienna, 1210 Vienna, Austria; 2 Department of Food and Resource Economics, University of Copenhagen, 1958 Frederiksberg C, Denmark; 3 Department of Veterinary and Animal Science, University of Copenhagen, 1870 Frederiksberg C, Denmark; 4 Department of Veterinary Clinical Science, Faculty of Health and Medical Sciences, University of Copenhagen, 1870 Frederiksberg C, Denmark; 5 School of Biodiversity, One Health and Veterinary Medicine, College of Medical, Veterinary and Life Sciences, University of Glasgow, Glasgow G61 1QH, UK

**Keywords:** internet, pet health information, medical advice, small animal practice, veterinarians, questionnaire-based survey

## Abstract

**Simple Summary:**

Owners of dogs, cats, and other companion animals increasingly make use of the internet to find out how to best care for their animals. This may affect owners’ relations with veterinarians in both positive and negative ways. A positive consequence could be that owners are better informed when they approach a veterinarian. However, there can also be challenging situations in which the owners may question veterinarians’ professional advice based on online information. Using a questionnaire, we found that a majority of Austrian, Danish, and UK veterinarians (*n* = 641) surveyed were occasionally confronted with clients who question their medical advice based on information obtained from the internet. In addition, the veterinarians were concerned about the potential for clients to misunderstand information found on the internet, or to develop unrealistic expectations of what is possible in small animal practices. As internet use becomes ever more widespread, we suggest that the types of resources that are available and used by animal owners should be further explored.

**Abstract:**

Veterinary medicine is increasingly affected by animal owners having the opportunity to become better informed on pet health issues by using various internet resources. Using an online questionnaire including a section on clients’ use of internet resources to obtain medical information, this study aimed to investigate veterinarians’ estimates of the percentage of clients using internet resources, how often clients question veterinarians’ professional medical advice based on online information, and veterinarians’ attitudes towards clients’ use of internet resources, across Austrian, Danish, and UK veterinarians (*n* = 641). The results show that 48.8% of respondents estimated that 40–79% of their clients use internet resources to find medical information. Further, 70–80% of respondents stated that they are occasionally challenged by clients questioning their advice based on online information. Although veterinarians recognized the potential advantages related to clients’ use of internet resources, such as an increased acceptance of advanced diagnostics and treatments, they also highlighted clients’ increased expectations or false impressions of small animal practices as potentially negative aspects in this context. As internet use increases, it seems likely that these issues will become increasingly important in the future.

## 1. Introduction

Internet access has become much more widely available in the last decade. In 2007, 55% of European Union households had internet access; by 2020, this had increased to 91% [1]. Against this background, the internet has become an indispensable part of people’s lives by providing, amongst other things, greater access to information and knowledge transfer. Veterinary medicine is also affected by these developments, as animal owners have the opportunity to learn more about pet health issues through unlimited access to veterinary medical information based on various internet resources including blogs, social media channels, veterinary associations, or practice websites. As owners become better informed, this will likely impact how they engage in dialogue with their veterinarian and can influence the decision making around diagnostic and therapeutic interventions.

In relation to this, Kogan and colleagues surveyed UK pet owners and found that there had been an increase in owners’ use of internet resources to obtain medical information [2]. Similarly, a survey among US pet owners conducted by the coalition Partners for Healthy Pets indicated that the percentage of pet owners searching for medical information on the internet due to their animal being sick or injured increased from 39% to 48% between 2010 and 2014 [3].

While clients’ interests in seeking medical information can be viewed as a positive development potentially improving their knowledge and understanding of patient care, other studies have shown that veterinarians are also concerned about the potential negative effects [2,4,5,6,7]. For example, online resources can distribute incorrect information; conversely, correct information can be misinterpreted by owners. The use of online resources may also inspire clients to try to treat their pets themselves, or to delay taking them to the veterinarian, thereby causing them harm. This has been highlighted in an Austrian focus group study among small animal veterinarians, who were increasingly confronted with clients who had misdiagnosed their pets based on information found online [8].

In addition, veterinarians increasingly experience situations where clients challenge their medical advice based on information they have obtained from the internet [3,5,8,9]. Niedziela published a statement with the title “British Vets Forced to Compete With ‘Dr. Google’” in which he reported that “nearly all British veterinarians say their clients’ behaviour was swayed by what the pet owners found online.” [3] As a consequence, veterinarians may increasingly have to compete with ‘Dr. Google’ [3,4] and hence may feel challenged in their professional authority.

Clients’ use of the internet to obtain medical information as well as information about specific veterinary services will undoubtedly increase [2,6]. Previous empirical studies have mainly focused on potential problems in relation to clients’ use of internet resources, and little is currently known about the potential benefits. The possible positive effects of clients becoming better informed include improved dialogue and understanding of their pets’ condition, as well as a greater acceptance of appropriate advanced diagnostic and treatment options.

Although a general increase in the use of internet resources can be seen in all European Union households, national differences exist that might influence the use of the internet in the veterinary context. For instance, in 2020, in the UK and Denmark, 94% of the population used the internet on a daily basis [10,11], in contrast to only 75% of the population in Austria [12]. It is likely that these differences will be reflected in the percentage of clients who use the internet to search for medical information, and the frequency with which clients question veterinarians’ advice. They may also impact veterinarians’ attitudes towards ‘Dr. Google’, both positively and negatively.

Further, it can be assumed that veterinarians’ attitudes will not only be influenced by socio-demographic and practice-specific factors such as age, employment status (self-employed versus employed), or the type of practice (independently owned versus corporate-owned), but potentially also by any previous negative experiences with clients who come to the practice with information they have obtained from the internet.

Therefore, the main aim of this study was to answer the following research questions: (i) What percentage of their clients do veterinarians estimate use internet resources to find medical information prior to consultation? (ii) How often do clients question veterinarians’ professional medical advice based on information obtained from the internet? (iii) What are veterinarians’ attitudes towards their clients’ use of internet resources, and what influences these attitudes? Throughout, we highlight the main similarities and differences between veterinarians in the three countries.

## 2. Materials and Methods

### 2.1. Study Population and Recruitment of Participants

The present study forms a part of a larger body of work on different aspects in modern small animal practices including investigations of how veterinarians manage conflicting ethical concerns during decision-making processes [13], the option of pet health insurance for dogs and cats [14], and the use of Facebook for veterinary practices [15].

In cooperation with small animal associations in Austria (VÖK), Denmark (DVA), and the UK (BSAVA), the link to the online-questionnaire was sent to 1195 Austrian, 1287 Danish, and 5138 UK veterinarians who worked (mainly) with small animals. For Denmark and Austria, data were collected from 2 March until 9 April 2020. Due to the COVID-19 pandemic outbreak and related organizational challenges, the BSAVA sent out the survey to its members at a later date and it was open between 30 March and 7 May 2020. Reminder e-mails were sent two weeks after opening the survey. The study received ethical approval from the Research Ethics Committee of SCIENCE and HEALTH at the University of Copenhagen (ReF: 504-0114/19-5000).

Since not all small animal veterinarians are members of the relevant veterinary associations in each country, this recruitment method introduces coverage error. Based on comparison with statistics on the study population, the assumed coverage error is 30% (*n* = 495) in Austria and 35.5% (*n* = 2812) in the UK. No specific data were available on the actual number of small animal veterinarians in Denmark, but as 90% are members of the DVA, the coverage error is likely around 10%.

### 2.2. Study Participants and Representativity of the Samples

A total of 829 veterinarians clicked on the survey weblink, and 773 (93.2%) continued on to answer at least one question [13]. Of the 773 responses, 132 questionnaires were excluded due to missing data specific to the analyses of clients’ use of internet resources (missing responses in Section B.4.1 ‘Client’s use of internet resources’—see Supplementary File S1). With a 15.9% (132/829) dropout-rate, the final sample included in the present study comprised 641 veterinarians, further consisting of 101 Austrian veterinarians (15.8%), 172 Danish veterinarians (26.8%), and 368 veterinarians from the UK (57.4%). The response rate was 8.5% for Austria, 13.3% for Denmark, and 7.2% for the UK. The varying sample size is mainly explained by the fact that the pool of veterinarians available to participate in the study varied between the three countries (as laid out in Section 2.1). Detailed information about socio-demographic and practice-specific factors for the whole study population, and for each sub-population, is listed in Supplementary File S2.

Since relevant census data were not available for Denmark, non-response analyses were conducted for Austria and UK only to identify whether the samples deviated from the population of small animal veterinarians [13]. For Austria, a relatively modest deviation was found based on a comparison of the geographical location. In this case, slightly more veterinarians from West Austria and slightly fewer veterinarians from East Austria participated in the study [13]. For the UK, we identified an overrepresentation of veterinarians in the ≤25–30-year group, and an underrepresentation in the 31–40-year group, compared to the study population [13].

### 2.3. Survey Design and Development

Items related to clients’ use of internet resources to obtain medical information were developed based on results of an Austrian focus group study [8] and a literature review of mainly empirical studies related to this issue [2,3,4,5,6,7]. The questionnaire was developed in English, and a two-step back-translation procedure was then used to produce the Austrian and Danish version of the questionnaire [13]. Further, the questionnaire underwent two stages of pre-testing. In the first step, cognitive interviews [16,17] were conducted with five Austrian veterinarians. In the second step, an online pre-test phase was conducted with ten Austrian, nine Danish, and six small animal veterinarians from the UK. Relevant feedback that helped to improve the quality of the data were considered and incorporated in the final versions of the questionnaire [13].

### 2.4. Survey Measures

In total, the questionnaire consisted of three sections (Supplementary File S1) and was designed so that questions could be skipped, enabling respondents to progress even if not all questions were completed in a previous section. Here, a detailed description is only provided for items in the two sections that give insights into the issue of clients’ use of internet resources to obtain medical information.

The first section, A, included 14 closed-ended questions on socio-demographic and practice-specific aspects. In the second section, B, there were three questions including one matrix with six statements related to clients’ use of internet resources. The first question asked veterinarians to estimate how many of their clients use internet resources to find medical information prior to a consultation. Answer options ranged from “None”, over “1–19%”, “20–39%”, and so on up to “80–100%”. The answer option “I don’t know” was also available. Further, six statements were presented to explore veterinarians’ attitudes towards their clients’ use of internet resources by focusing on possible positive (e.g., improved discussions due to clients’ greater knowledge) and negative effects (e.g., creating the wrong impression of modern small animal practices). Respondents could indicate their level of agreement with each statement through a 7-point Likert scale [18]: 1 “Strongly disagree”, 2 “Disagree”, 3 “Somewhat disagree”, 4 “Neutral (neither agree nor disagree), 5 “Somewhat agree”, 6 “Agree”, and 7 “Strongly agree”. Further, an “I don’t know” option was provided. The third question asked veterinarians how often clients questioned their professional advice based on information they obtained from internet resources. Response options were “Never”, “Occasionally”, “Frequently”, “Always”, and “I don’t know”.

In addition, section B included a question about several factors that could influence clients’ desire to further pursue therapy beyond veterinarians’ professional recommendations, including the factor that “Medical information obtained using internet resources” influences clients’ desire to pursue further therapy beyond veterinarians’ recommendation. Possible answer options were “Not at all”, “Slightly”, “Moderately”, “Relatively Strong”, and “Strong”. The answer option “I don’t know” was also provided.

### 2.5. Data Analysis

All three online surveys were set up using the survey software Alchemer^®^ (Alchemer^®^, Louisville, KY, USA). IBM^®^ SPSS^®^ Statistics version 27.0 (IBM^®^ SPSS^®^ Statistics, Chicago, IL, USA) was used for all analyses. Univariate descriptive statistics were presented in tables, figures, or text. For bivariate analysis, Chi-square tests or Kruskal–Wallis H tests were conducted to test whether the frequency distribution differed between the Austrian, Danish, and UK sub-populations. The significance level was 0.05.

Ordinal regression analyses were conducted to identify the extent to which socio-demographic and practice-specific factors, clients’ use of internet resources, percentage of clients who question veterinarians’ professional advice, and the impact of internet resources on clients’ desire to pursue treatment had an effect on veterinarians’ attitudes towards clients’ use of internet resources (Supplementary File S3). For each country, six ordinal regression analyses were performed in which statements were inserted as dependent variables. The answer option “I don’t know” was excluded from these analyses. Categorical variables inserted in the regression analyses were gender (1 = male; 2 = female), type of practice (1 = independently owned; 2 = corporate-owned), employment status (1 = self-employed; 2 = employed), percentage of clients using internet resources (1 = None–19%; 2 = 20–59%; 3 = over 60%), and frequency of situations in which clients question veterinarians advice based on information obtained from internet resources (1 = ”Never”; 2 = ”Occasionally”; 3 = ”Frequently/Always”). Since no veterinarian from Austria or Denmark and only two veterinarians from the UK indicated that this happened “always”, the answer options “frequently” and “always” were combined for these analyses. Age (range: 23–83 years) and the impact of internet resources on clients’ desire to pursue further therapy beyond that recommended were included as continuous variables. By employing a variance inflation factor (VIF) above 5.0 as an indicator of multicollinearity, VIF statistics suggested that age and working experience should not be included as continuous variables in the same models (VIF age: for Austria between 7.781 and 8.346, for Denmark between 18.006 and 17.813, and for UK between 17.813 and 18.026; VIF working experience: for Austria between 6.885 and 7.096, for Denmark between 17.620 and 17.732, and for UK between 17.506 and 17.732). In order to avoid multicollinearity, we chose to use only veterinarians’ age in the regression analyses, since age in particular seems to be the important factor with respect to familiarity with and use of the internet, which might therefore influence their attitudes towards clients’ use of internet resources in the veterinary context. The answer option “Other” for type of practice and employment status, and the answer option “I don’t know” for the impact of internet resources on clients’ desire to pursue further therapy, percentage of clients using internet resources, and frequency of situations in which clients question veterinarians’ advice, were excluded from these analyses. Further, since only two Austrian veterinarians indicated that they worked in a corporate-owned practice, the variable business type was excluded for all six ordinal regression models run for Austria.

## 3. Results

### 3.1. Veterinarians’ Estimates of Clients Use of Internet Resources Prior to Consultation

Table 1 presents the veterinarians’ estimates of how many of their clients used internet resources to find medical information prior to a consultation. Almost half of the respondents (48.8%) estimated that between 40–79% of their clients used internet resources to find medical information prior to their veterinary consultation. No significant difference was identified between Austrian, Danish, and UK veterinarians (H (2) = 2.300, *p* = 0.301).

### 3.2. Percentage of Clients Questioning Veterinarian’s Professional Medical Advice Based on Information from the Internet

**Figure 1:**
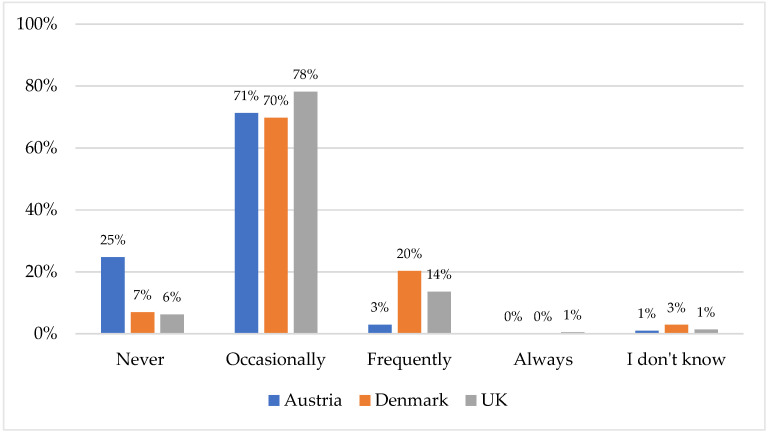
Percentage of clients questioning veterinarians’ professional advice based on information obtained from the internet for Austria (*n* = 101), Denmark (*n* = 172), and the UK (*n* = 368).

In all three countries, between 70–78% of the respondents indicated that their clients occasionally questioned their professional advice based on information they had obtained from the internet (Figure 1). Significant differences were identified between the three countries (H (2) = 37.111; *p* < 0.001). Significantly more Austrian veterinarians indicated that they were ‘never’ confronted with clients who questioned their advice compared to Danish (χ^2^ (1) = 12.487; *p* < 0.001) and UK (χ^2^ (1) = 23.925; *p* < 0.001) veterinarians. In comparison, significantly more veterinarians from Denmark and the UK indicated that they were ‘frequently’ confronted with such clients, in contrast to their Austrian colleagues (DK: χ ^2^ (1) = 28.533, *p* < 0.001; UK: χ^2^ (1) = 27.091, *p* < 0.001).

### 3.3. Veterinarians’ Attitudes towards Clients’ Use of Internet Resources

Six statements were presented exploring veterinarians’ attitudes towards clients’ use of internet resources to obtain medical information (Table 2). In general, veterinarians from all three countries agreed that the use of internet resources increased both clients’ expectations and acceptance of advanced diagnostics and treatments.

However, significant differences were identified between the countries. Danish respondents significantly more frequently agreed that clients’ use of internet resources led to a “greater acceptance of advanced diagnostic and treatments” and less frequently agreed that it “creates the wrong impression of modern small animal practice” compared to their Austrian (*p* = 0.007; *p* = 0.047) and UK (*p* < 0.001; *p* < 0.001) colleagues. The opinion that clients’ use of internet resources “improves discussion about diagnostic and treatment options as clients have a greater knowledge” obtained less agreement from Austrian veterinarians compared to their colleagues from Denmark (*p* = 0.001) and the UK (*p* = 0.017). Further, UK veterinarians more frequently agreed with the statement that the use of internet resources “causes clients to form strong opinions so that veterinarians have to justify their diagnostics and therapeutic steps” than Austrian (*p* = 0.002) and Danish (*p* < 0.001) veterinarians.

### 3.4. What Explains Veterinarians’ Attitudes towards Clients’ Use of Internet Resources?

Six ordinal regression models were run for each country to try to understand the attitudes underpinning the answers to the statements listed in Table 2 (Supplementary File S3). The attitudes were particularly impacted by the number of clients that the veterinarians thought used internet resources to obtain information, and the frequency with which they were questioned or challenged by clients using information from such sources. A detailed overview of the significant findings is provided below.

#### 3.4.1. Statement: Clients’ Use of Internet Resources Results in Greater Acceptance of Advanced Diagnostics and Treatments

In Austria and the UK, veterinarians who indicated that their professional advice was ‘frequently/always’ questioned by clients were less likely to agree with the statement, compared to veterinarians who indicated that they were ‘never’ (Austria: χ^2^ (1) = 11.009, *p* < 0.001) or only ‘occasionally’ (Austria: χ^2^ (1) = 8.578, *p* = 0.003; UK: χ^2^ (1) = 6.940, *p* = 0.008) questioned by clients.

In the UK, the likelihood of agreeing with this statement increased with age (UK: χ^2^ (1) = 12.210, *p* < 0.001). In addition, the veterinarians who estimated that ‘none–19%’ of their clients make use of internet resources were less likely to agree with the statement compared to those veterinarians who estimated that over 60% of their clients use internet resources (UK: χ^2^ (1) = 4.679, *p* = 0.031).

For Denmark, male veterinarians were more likely to agree with the statement than their female colleagues (DK: χ^2^ (1) = 5.411, *p* = 0.020).

#### 3.4.2. Statement: Clients’ Use of Internet Resources Results in Greater Expectations of Advanced Diagnostics and Treatments

For the UK and Denmark, the likelihood of agreeing with this statement increased with age (DK: χ^2^ (1) = 4.684, *p* = 0.030; UK: χ^2^ (1) = 5.870, *p* = 0.015).

Further, UK veterinarians who agreed more strongly that internet resources impact clients’ desire to pursue treatment were more likely to agree that clients have greater expectations of advanced diagnostics and treatments (UK: χ^2^ (1) = 21.159, *p* < 0.001). In addition, UK veterinarians who stated that their professional advice is ‘never’ (UK: χ^2^ (1) = 12.945, *p* < 0.001) or only ‘occasionally’ (UK: χ^2^ (1) = 12.525, *p* < 0.001) questioned by clients were less likely to agree with the statement, compared to their colleagues who are ‘frequently/always’ questioned by clients.

#### 3.4.3. Statement: Clients’ Use of Internet Resources Improves the Discussion about Diagnostic and Treatment Options as the Clients Have Greater Knowledge

For the UK, female professionals were more likely to agree with this statement than their male colleagues (χ^2^ (1) = 4.9730, *p* = 0.026). In addition, the likelihood of agreeing with the statement increased with age (χ^2^ (1) = 16.835, *p* < 0.001).

#### 3.4.4. Statement: Clients’ Use of Internet Resources Causes Clients to Form Strong Opinions So That Veterinarians Have to Justify Their Diagnostic and Therapeutic Steps

In all three countries, the likelihood of agreeing with the statement decreased with an increasing age (Austria: χ^2^ (1) = 4.003, *p* = 0.045; DK: χ^2^ (1) = 5.7111, *p* = 0.017; UK: χ^2^ (1) = 7.828, *p* = 0.005). Further, Austrian, Danish, and UK veterinarians who indicated that their professional advice is ‘frequently/always’ questioned by clients were more likely to agree with the statement compared to colleagues who indicated that clients ‘never’ questioned their advice (Austria: χ^2^ (1) = 5.251, *p* = 0.022; Denmark: χ^2^ (1) = 13.798, *p* < 0.001; UK: χ^2^ (1) = 13.886, *p* < 0.001).

Danish and UK veterinarians who stated that their professional advice was ‘occasionally’ questioned by clients were less likely to agree with the statement compared to veterinarians who indicated that this happened ‘frequently/always’ (Denmark: χ^2^ (1) = 4.996, *p* = 0.025; UK: χ^2^ (1) = 8.228, *p* = 0.004).

In addition, Austrian veterinarians who indicated that over 60% of their clients used internet resources were more likely to agree with this statement compared to those who estimated the level to be ‘none–19%’ (χ^2^ (1) = 4.561, *p* = 0.033) or ‘20–59%’ (χ^2^ (1) = 7.376, *p* = 0.007). Further, in Denmark and the UK, the veterinarians who indicated that the use of internet resources had a strong impact on clients’ desire to pursue treatment were more likely to agree with this statement (Denmark: χ^2^ (1) = 11.551, *p* < 0.001; UK: χ^2^ (1) = 4.216, *p* = 0.040).

#### 3.4.5. Statement: Clients’ Use of Internet Resources Creates a False Impression of Modern Small Animal Practices

In Austria, female veterinarians were more likely to agree with this statement than their male colleagues (χ^2^ (1) = 6.068, *p* = 0.014). Additionally, veterinarians who indicated that over 60% of their clients use internet resources were more likely to agree with this statement than those veterinarians who indicated that ‘none–19%’ of their clients use them (χ^2^ (1) = 6.803, *p* = 0.009). 

In the UK, veterinarians who indicated that their professional advice was ‘never’ (χ^2^ (1) = 17.682, *p* < 0.001) or only ‘occasionally’ questioned (χ^2^ (1) = 4.001, *p* = 0.045) were less likely to agree with this statement compared to their colleagues who were ‘frequently/always’ questioned by clients. Further, UK veterinarians who indicated that internet resources had a strong impact on clients’ desire to pursue treatment were more likely to agree with this statement (χ^2^ (1) = 6.080, *p* = 0.014).

## 4. Discussion

The results of this comparative study show that veterinarians in all three countries gave a similar estimate of the number of clients that consult the internet prior to a consultation with their veterinarian. Approximately half of the Austrian, Danish, and UK respondents estimated that between 40–79% of their clients used the internet. As there is a less frequent use of the internet in Austria in general [10,11,12], we expected that Austrian veterinarians might indicate that a lower percentage of their clients make use of internet resources compared to their Danish and UK colleagues, but this was not the case. A possible explanation for this might be that animal owners are specifically motivated to look for information about their animal’s treatment, irrespective of their general daily use of the internet. 

This would seem to be supported by the results of Kogan and colleagues [2], who found that 94% of surveyed US animal owners used the internet to find medical information, although at the time of data collection (2009), it was estimated that only 76% of American adults in general used the internet [19]. Interestingly, even though a study among UK veterinarians indicated that, compared with our study results, more veterinarians (68%) believed that 41–80% of their clients used the internet to look for health information about their animal [5], veterinarians’ estimates in both studies are clearly lower than the proportion of owners that have been found to use internet resources. For example, in a US study, 94% of the surveyed animal owners indicated that they used the internet to find medical information [2]. This difference may be explained by the fact that not all clients necessarily tell their veterinarian that they have consulted the internet for information. This would seem to be the case based on Kogan and colleagues [5], who reported that only 15% of the surveyed UK veterinarians think that the majority (61–100%) of clients discuss the animal health information obtained online with their veterinarian. Therefore, we recommend that future studies attempt to identify the actual number of animal owners who make use of the internet in the veterinary context.

If we now consider why clients might use the internet to seek information on their animals’ care, Kogan and colleagues proposed two main reasons: firstly, animal owners may wish to be more informed or seek clarification following a discussion with the veterinarian [2]. Second, they may not believe or may disagree with the information provided by the veterinarian [2]. In the latter case, this can lead to veterinarians being confronted by clients who question their professional advice. Thus, a further aim of our study was to identify how often veterinarians are confronted with clients who question their medical advice based on information they have obtained online. We found that 70–78% of Austrian, Danish, and UK respondents ‘occasionally’ found themselves in such a situation. However, significantly more UK and Danish veterinarians stated that they are ‘frequently’ confronted by clients who question their medical advice compared to their Austrian colleagues. A possible explanation for the observed differences could be that Austrian veterinarians may be underestimating the number of clients who challenge them with information that has been obtained online. However, a more likely explanation is that many more relevant or informative websites were available to clients in the UK and Denmark, which may have led to a higher frequency of UK and Danish clients questioning the veterinarians’ advice. Finally, it may be the case that Austrian animal owners wish to be better informed and prepared for veterinary consultations, but simply choose not to subsequently question their veterinarians’ advice. Although consultations in which clients question veterinarians’ medical advice may be experienced as being rather challenging, it is important that veterinarians do not discourage clients from asking questions, irrespective of the source of the information. Only through such open discussion can clients’ concerns be acknowledged, and a situation of shared-decision making be encouraged, rather than one in which the client feels pressured to accept the veterinarians’ advice.

However, there is no doubt that when clients question their veterinarians’ medical advice, it can affect their relationship. In a survey of 100 veterinarians in the UK, Kogan and colleagues reported that 54% of respondents felt that clients’ use of the internet negatively impacted the relationship, and only 35% said that it had a positive effect [5]. In addition, 40% of veterinarians thought that clients obtaining medical information from the internet had a negative impact on the health of the patient, 37% thought it had a positive effect, and 23% stated it had no effect [5]. In this context, a further aim of our study was to gain more detailed insights into veterinarians’ attitudes towards the potential positive and negative effects of clients’ use of internet resources.

If we first consider the possible positive effects, we assumed that the use of internet resources would improve discussions between veterinarians and clients about diagnostic and treatment options, as the clients would be better informed. We found that Austrian veterinarians significantly more often disagreed with this than their Danish or UK colleagues. This might be explained by the findings of Springer and colleagues, who reported that Austrian small animal veterinarians indicated that clients’ ability to understand medical issues during the consultation dialogue varied widely, and that there is a need to deal flexibly with clients [8]. Yet, we would expect this to apply equally across countries, as the ability of clients to understand medical information varies greatly, irrespective of whether that information is provided by the veterinarian or independently sourced online. In the latter case, client understanding can be complicated if the information obtained online is erroneous, or if valid information is misinterpreted. This is supported by Kogan and colleagues, who identified that 73% of their surveyed veterinarians believed that few (i.e., 0 to 40%) of their clients understood what they read online [5]. If this concern is shared more widely, it could explain why respondents from all three countries mainly disagreed that clients’ use of the internet leads to the clients being better informed than the veterinarian. Therefore, further work is required to examine the quality of existing online resources, how clients access them, and whether this varies between countries.

In addition to improving dialogue, we had also expected that clients’ use of internet resources might increase their acceptance of advanced diagnostic and treatments options. In general, veterinarians from all three countries tended to agree with this positive effect. Interestingly, Austrian and UK veterinarians who indicated that their medical advice is never questioned by clients were more likely to agree with this statement compared to colleagues who are frequently or always questioned by their clients. This is not surprising, as it can be assumed that veterinarians who are often challenged based on online information might feel this outweighs any benefits from clients’ greater acceptance of advanced treatments. Despite the overall agreement, we identified that one quarter of the respondents had a rather neutral stance, indicating neither a clear agreement nor disagreement towards this statement. A possible explanation, based on the work by Kogan and colleagues, is that while some owners might feel eager, confident, or reassured by the information they obtain online, the amount of available information can also be frustrating, confusing, or even overwhelming for others [2]. This can lead to the uncertainty of the clients, rather than an increased willingness to accept the advanced diagnostics and treatments suggested by their veterinarians. Further, clients need to be able to understand and critically review the information that is available online in order to use it to appropriately inform their decisions, and the ability to do so will vary with their background and educational level. For example, Kogan and colleagues found that clients with at least some college education were more likely to visit recommended websites compared to clients with a high school diploma or general educational development, which might result in a greater acceptance of advanced treatments [6].

Further, our study revealed some differences between male and female respondents. For example, female veterinarians from the UK were more likely to agree that clients’ use of internet resources improves discussions about diagnostic and treatment options, as the clients are more knowledgeable. Recent research on the differences between women and men related to the use of internet resources in a veterinary context mainly focusses on the clients’ perspectives [2]. To the best of the authors’ knowledge, no comparable data exist investigating potential gender differences based on studies of veterinarians. However, in the human medical field, a survey of Dutch rheumatologists and oncologists by Uden-Kraan and colleagues reported that female physicians more often experience patients raising information they have obtained from the internet during a consultation compared to their male colleagues [20]. Based on that, Uden-Kraan and colleagues concluded that female physicians might be considered more approachable by patients and more open to the use of internet resources [20], which can improve discussion during a consultation. This might also be the case in the veterinary context, as in general, female veterinarians were more likely to agree that internet knowledge improved discussions with their clients.

However, as previously described, Kogan and colleagues identified that veterinarians thought that the use of the internet also had a negative impact on the relationship between the client and the veterinarian, as well as on the health status of the patient [5]. In addition, the results of an Austrian focus group study among small animal veterinarians highlighted professionals’ concerns about increasing client expectations in regard to advanced technology in diagnosis and treatment, as well as about the increasing use of social media platforms to exchange with other clients via the internet [8]. The results of our study support this, with veterinarians from all three countries agreeing that the use of internet resources increased client expectations regarding advanced diagnostics and treatments. While a certain level of expectation on the clients’ side can be beneficial when it comes to dialogue about the use of advanced diagnostic tests or treatments during patient care, it can be challenging when clients’ expectations differ significantly from those of the veterinarian, or from the patients’ best interests [8]. This was highlighted in our study, where UK veterinarians who thought that internet resources had a strong impact on clients’ desire to pursue treatment against the veterinarians’ advice, or ‘frequently’ or ‘always’ experienced clients questioning their medical advice, were more likely to agree that the internet leads to increased client expectations.

Better-informed clients may also form strong opinions and expect veterinarians to justify their diagnostic and therapeutic steps. Interestingly, we found that in all three countries, younger veterinarians were more likely to view this as a negative effect. This might be explained in two ways: Firstly, clients may behave differently towards younger and less experienced veterinarians compared to older and more experienced professionals, more often challenging the younger veterinarians to justify their treatments. Alternatively, younger and less experienced veterinarians may feel more insecure when facing owners with strong opinions, feeling that they have to justify themselves, whereas older and more experienced colleagues might feel less stressed and more secure in dealing with such clients.

Finally, the volume and nature of information on the internet can sometimes give clients an unrealistic impression of modern small animal practices. UK veterinarians were more likely to agree with this compared to Austrian and Danish veterinarians, perhaps because of the high media presence of cutting-edge veterinary medicine within the UK [21]. For example, Noel Fitzpatrick is a well-known veterinary surgeon who has a very popular television series (‘Supervet’) featuring challenging cases receiving advanced treatments [21,22]. He also has a very visible presence on various internet channels such as YouTube and other social media platforms, and this may indirectly influence the information about possible therapeutic options on other websites. Clients can be highly influenced by exposure to such information and gain a false impression of the types of services available in most veterinary practices. UK veterinarians in particular believe that internet resources have a strong impact on clients’ desire to pursue treatment and are more likely to agree that it creates a false impression of modern small animal practice than vets in Austria and Denmark. Therefore, we recommend that veterinarians should proactively direct clients towards appropriate and factually accurate online resources where they can seek further information if desired. In this context, Kogan and colleagues found that of the 94 veterinarians they surveyed who suggested websites to clients, 32% verbally recommended particular websites, 21% gave written recommendations, and 17% gave both written and verbal recommendations [5]. By providing both verbal and written information, as well as recommending reliable websites, veterinarians should be able to manage clients’ unrealistic impressions of modern small animal practices.

Although this study involves three countries to enable a comprehensive investigation of our research questions, the study is subject to limitations: 

First, since not all small animal veterinarians are members of a small animal association, the study is subject to a selection bias, especially in Austria and the UK, with a coverage error of around 30%. In addition, even though there was a very good socio-demographic coverage by the three samples, the rather low response rates may lead to potential non-response bias, where participants deviate systematically from those invited respondents who did not reply. Further, the number of participants from the three countries varied, with a higher number of UK veterinarians and much smaller numbers from Denmark and Austria. This may have influenced the identification of significant differences in our regression analyses. In Austria, the higher proportion of older (50–59 years) and self-employed veterinarians and the lower proportion of veterinarians with 0.5–5 years of work experience compared to Denmark and UK could have influenced the differences we identified. Further, of the 21 veterinarians who were categorized as “Other” in relation to business type, only three indicated that they were retired (one from each country). Due to the small number and mostly short duration of retirement (<0.5, 1 and 7 years), we did not consider this aspect in our analyses in the context of their ability to recall information relating to their clients and clients’ use of internet resources.

Second, to generate an overview of the different aspects that are of relevance in relation to clients’ use of internet resources, we formulated six statements to identify veterinarians’ attitudes toward this issue based on the results of an Austrian focus group study [8] as well as the existing literature including empirical studies and anecdotal knowledge [2,3,4,5,6,7]. However, it was not specified whether veterinarians should answer the statements by referring to their own personal experiences, or in relation to the profession in general. These aspects should be explored independently in future research.

Third, the use of answer options such as “occasionally” or “frequently” is more subjective compared to, for example, a specific percentage. Thus, the respondents may have interpreted these terms differently, introducing variability into the reported occurrence rates.

Fourth, veterinarians were asked how many of their clients they thought used internet resources prior to a consultation, which may not reflect the actual number who do so, as not all clients will explicitly share that information with their veterinarians. Future work should collect that information directly from clients for a comparison.

Fifth, when we asked veterinarians about their perceptions of clients’ acceptance or otherwise of ‘advanced’ diagnostics and treatments, we did not provide a detailed description of the term “advanced”. Although a generally understood and commonly used term within the profession, it may have been interpreted in different ways by the respondents, perhaps depending on their working background and/or level of specialization.

Although our results indicate how often veterinarians are challenged by clients questioning their professional advice based on information obtained from the internet, we did not specify the context, e.g., clients questioning veterinarians on diagnostic or treatment recommendations, costs, vaccination policies, behavioral issues, etc. Further, we did not ask how often veterinarians showed interest in what kinds of information clients have found on the internet. Hence, we recommend that these aspects should be explored in future studies.

## 5. Conclusions

We conclude that veterinarians generally recognize the advantages of clients’ use of internet resources to become better informed, for example, with respect to making them potentially more accepting of advanced diagnostics and treatments in patient care. However, it can also have a negative impact if clients misunderstand the information or develop unrealistic expectations of what is possible in most small animal practices. As internet use increases, and the available information continues to rapidly expand, such concerns will play an increasing role in modern veterinary practices. Thus, further research is recommended to understand both the attitudes of clients towards internet resources and the types of resources most frequently used to search for veterinary medical information.

## Figures and Tables

**Table 1:** Veterinarians’ estimates of clients’ use of internet resources to obtain medical information prior to consultation for the whole study population and by country.

	All Countries (*n* = 600)	Austria (*n* = 87)	Denmark (*n* = 160)	UK (*n* = 353)	Test *
None	1 (0.2)	0.0 (0.0)	1 (0.6)	0.0 (0.0)	H (2) = 2.300 *p* = 0.301
1–19%	74 (12.3)	10 (11.5)	28 (17.5)	36 (10.2)	
20–39%	144 (24.0)	25 (28.7)	34 (21.3)	85 (24.1)	
40–59%	150 (25.0)	16 (18.4)	40 (25.0)	94 (26.6)	
60–79%	143 (23.8)	24 (27.6)	34 (21.3)	85 (24.1)	
80–100%	57 (9.5)	9 (10.3)	14 (8.1)	34 (9.6)	
I don’t know	31 (5.2)	3 (3.4)	9 (5.4)	19 (5.4)	

Count (%). * Kruskal–Wallis H test (answer option ‘I don’t know’ was excluded from this analysis).

**Table 2:** Veterinarians’ attitudes towards clients’ use of internet resources.

Nr.	Clients’ Use of Internet Resources…		Austria (*n* = 96–100)	Denmark (*n* = 169–172)	UK (*n* = 357–367)	Test *
1	▪ results in greater acceptance of advanced diagnostics and treatments.	Disagreement (1–3)	19 (19.6)	7 (4.1)	53 (14.8)	H (2) = 16.688, *p* < 0.001
		Neutral (4)	25 (25.4)	47 (27.8)	100 (27.9)	AT vs. DK: *p* = 0.007 ^a^
		Agreement (5–7)	53 (54.7)	115 (68.0)	205 (57.2)	AT vs. UK: *p* = 1.00 ^a^
		Mean ± Std.	4.57 ± 1.18	4.98 ± 1.07	4.52 ± 1.51	DK vs. UK: *p* < 0.001 ^a^
2	▪ results in greater expectations of advanced diagnostics and treatments	Disagreement (1–3)	13 (13.0)	22 (12.9)	25 (7.0)	H (2) = 9.917, *p* = 0.007
		Neutral (4)	15 (15.0)	41 (24.0)	45 (12.5)	AT vs. DK: *p* = 1.00 ^a^
		Agreement (5–7)	72 (72.0)	198 (63.2)	289 (80.5)	AT vs. UK: *p* = 0.131 ^a^
		Mean ± Std.	5.00 ± 1.20	4.95 ± 1.2	5.25 ± 1.12	DK vs. UK: *p* = 0.012 ^a^
3	▪ improves the discussion about diagnostic and treatment options as the clients have greater knowledge.	Disagreement (1–3)	44 (44.0)	40 (22.5)	105 (28.6)	H (2) = 13.976, *p* = 0.001
		Neutral (4)	22 (22.0)	39 (2 3.1)	75 (20.4)	AT vs. DK: *p* = 0.001 ^a^
		Agreement (5–7)	34 (34.0)	92 (54.4)	187 (51.0)	AT vs. UK: *p* = 0.017 ^a^
		Mean ± Std.	3.87 ± 1.44	4.53 ± 1.32	4.29 ± 1.38	DK vs. UK: *p* = 0.255 ^a^
4	▪ can lead to situations where clients are better informed than I am.	Disagreement (1–3)	68 (68.0)	81 (57.6)	197 (53.7)	H (2) = 5.474, *p* = 0.065
		Neutral (4)	19 (19.0)	25 (14.5)	62 (16.9)	
		Agreement (5–7)	13 (13.0)	48 (27.9)	108 (29.4)	
		Mean ± Std.	3.02 ± 1.30	3.31 ± 1.53	3.41 ± 1.45	
5	▪ causes clients to form strong opinions so that veterinarians have to justify their diagnostic and therapeutic steps.	Disagreement (1–3)	18 (18.0)	34 (20.0)	37 (10.1)	H (2) = 26.953, *p* < 0.001
		Neutral (4)	11 (11.0)	15 (8.8)	31 (8.5)	AT vs. DK: *p* = 1.00 ^a^
		Agreement (5–7)	71 (71.0)	121 (71.1)	297 (81.4)	AT vs. UK: *p* = 0.001 ^a^
		Mean ± Std.	4.81 ± 1.36	4.76 ± 1.40	5.32 ± 1.31	DK vs. UK: *p* < 0.001 ^a^
6	▪ creates a false impression of modern small animal practices.	Disagreement (1–3)	24 (25.0)	58 (36.5)	62 (17.4)	H (2) = 56.893, *p* < 0.001
		Neutral (4)	35 (36.5)	56 (35.2)	66 (18.5)	AT vs. DK: *p* = 0.047 ^a^
		Agreement (5–7)	37 (38.6)	45 (28.4)	229 (64.2)	AT vs. UK: *p* = 0.002 ^a^
		Mean ± Std.	4.24 ± 1.51	3.75 ± 1.40	4.77 ± 1.37	DK vs. UK: *p* < 0.001 ^a^

Count (%). * Kruskal–Wallis H test. ^a^ Bonferroni correction was applied for multiple comparison between three countries and significant variables.

## Data Availability

All relevant data are within the paper and its Supplementary Materials.

## Supplementary Materials

The following supporting information can be downloaded at: https://www.mdpi.com/article/10.3390/ani12162117/s1, File S1: Questionnaire; File S2: Socio-demographic and practice-specific data; File S3: Ordinal regression analyses.
