# Supplementary material for: Compete or Cooperate with ‘Dr. Google’? Small Animal Veterinarians’ Attitudes towards Clients’ Use of Internet Resources—A Comparative Study across Austria, Denmark and the UK
Source: Animals (Basel). 2022 Aug 18;12(16):2117. doi: 10.3390/ani12162117 (PMC9404757; doi:10.3390/ani12162117)
Supplement: Supplementary file 1 [file animals-12-02117-s001.zip › Supplementary Files/2_Sociodemographic_Data.pdf]

Supplementary File S2:

Socio-demographic and practice-specific factors for the whole study population and each sub-population from Austria, Denmark and UK

	All countries (N=608-641)	Austria (n=94-101)	Denmark (n=161-172)	United Kingdom (n=353-368)	Tests
**GENDER**					
Male	179 (28.1)	25 (25.0)	43 (25.0)	111 (30.5)	$\chi^2(2)=2.324$, $p=0.313$
Female	457 (71.9)	75 (75.0)	129 (75.0)	253 (69.5)	
**AGE (in years)**					
23-29	86 (14.1)	5 (5.3)	16 (9.9)	65 (18.4)	$H(2)=4.877$, $p=0.087$
30-39	143 (23.5)	21 (22.3)	40 (24.8)	82 (23.2)	
40-49	145 (23.8)	27 (28.7)	40 (24.8)	78 (22.1)	
50-59	161 (26.5)	37 (39.4)	42 (26.1)	82 (23.2)	
≥ 60	73 (12.0)	4 (4.3)	23 (14.3)	46 (13.0)	
**WORK EXPERIENCE (in years)**					
0.5-5	135 (21.2)	11 (11.1)	39 (22.9)	85 (23.2)	$H(2)=0.740$, $p=0.691$
6-10	69 (10.8)	14 (14.1)	19 (11.2)	36 (9.8)	
11-20	132 (20.8)	29 (29.3)	32 (18.8)	71 (19.3)	
≥21	300 (47.2)	45 (45.5)	80 (47.1)	175 (47.7)	
**BUSINESS TYPE**					$\chi^2(2)=131.089$, $p<0.001$
Independently owned	370 (57.7)	96 (95.0)	131 (76.2)	143 (38.9)	AT vs. DK: $\chi^2(1)=14.254$, $p<0.001$[a]
Corporate owned	216 (33.7)	2 (2.0)	28 (16.3)	186 (50.5)	AT vs. UK: $\chi^2(1)=90.990$, $p<0.001$[a]
Other[b] (incl. university and shelter)	55 (8.6)	3 (3.0)	13 (7.6)	39 (10.6)	DK vs. UK: $\chi^2(1)=65.963$, $p<0.001$[a]
**EMPLOYMENT STATUS**					$\chi^2(2)=106.720$, $p<0.001$
Self-employed	226 (35.3)	80 (79.2)	62 (36.3)	83 (22.6)	AT vs. DK: $\chi^2(1)=45.496$, $p<0.001$[a]
Employed	393 (61.4)	20 (19.8)	105 (61.0)	268 (73.0)	AT vs. UK: $\chi^2(1)=107.088$, $p<0.001$[a]
Other[b] (retired and unspecified)	21 (3.3)	1 (1.0)	4 (2.3)	16 (4.4)	DK vs. UK: $\chi^2(1)=10.785$, $p=0.003$[a]

Counts (percentage)

[a] Bonferroni correction was applied for multiple comparison between three countries and significant variables

[b] Answer option "Other" were excluded from bivariate statistics
