# Supplementary material for: Compete or Cooperate with ‘Dr. Google’? Small Animal Veterinarians’ Attitudes towards Clients’ Use of Internet Resources—A Comparative Study across Austria, Denmark and the UK
Source: Animals (Basel). 2022 Aug 18;12(16):2117. doi: 10.3390/ani12162117 (PMC9404757; doi:10.3390/ani12162117)
Supplement: Supplementary file 1 [file animals-12-02117-s001.zip › Supplementary Files/3_Ordinal_regression_Analyses.pdf]

Supplementary File S3:
Ordinal regression analyses of socio-demographic and practice-specific factors on attitudes towards clients' use of internet resources

**Model 1 AUSTRIA: Clients' use of internet resources results in greater acceptance of advanced diagnostics and treatments** ($\chi^2(8)$=15.900, P=0.044)					
			Hypothesis Test		
	B	Std. Error	Wald Chi-Square	df	Sig.
Gender *(ref. cat.: female)*	1.240	0.6497	3.644	1	0.56
Percentage of clients using internet resources (1=None-19%) *(ref.cat.: over 60%)*	0.302	07690	0.154	1	0.695
Percentage of clients using internet resources (2=20-59%) *(ref.cat.: over 60%)*	-0.204	0.5442	0.141	1	0.707
Frequency of situations in which clients question vet's advice (1=Never) *(ref.cat.=Frequently/Always)*	5.844	1.7614	11.009	1	<0.001
Frequency of situations in which clients question vet's advice (2=Occasionally) *(ref.cat.=Frequently/Always*	5.058	1.7270	8.578	1	0.003
Employment type *(ref. cat.: employed)*	1.041	0.6960	2.235	1	0.214
age	-0.040	0.0321	1.542	1	0.214
Impact of internet resources on clients' desire to pursue therapy	0.088	0.2047	0.186	1	0.666
**Model 1 DENMARK: Clients' use of internet resources results in greater acceptance of advanced diagnostics and treatments.** ($\chi^2(9)$=17.604, P=0.040)					
Gender *(ref. cat.: female)*	1.137	0.4888	5.411	1	0.020

Percentage of clients using internet resources (1=None-19%) *(ref.cat.: over 60%)*	-0.635	0.5565	1.301	1	0.254
Percentage of clients using internet resources (2=20-59%) *(ref.cat.: over 60%)*	-0.044	3.992	0.012	1	0.913
Frequency of situations in which clients question vet's advice (1=Never) *(ref.cat.=Frequently/Always)*	0.873	0.7259	1.448	1	0.229
Frequency of situations in which clients question vet's advice (2=Occasionally) *(ref.cat.=Frequently/Always*	0.097	0.4520	0.046	1	0.830
Employment type *(ref. cat.: employed)*	0.614	0.4411	1.937	1	0.164
age	0.016	0.0181	0.806	1	0.369
Impact of internet resources on clients' desire to pursue therapy	-0.012	0.1501	0.007	1	0.935
Business type *(ref. cat.: corporate-owned)*	0.482	0.5016	0.922	1	0.337
**Model 1 UK: Clients' use of internet resources results in greater acceptance of advanced diagnostics and treatments.** ($\chi^2(9)$=26.522, P=0.002)					
Gender *(ref. cat.: female)*	-0.133	0.2799	0.227	1	0.031
Percentage of clients using internet resources (1=None-19%) *(ref.cat.: over 60%)*	-0.927	0.4287	4.679	1	0.031

Percentage of clients using internet resources (2=20-59%) *(ref.cat.: over 60%)*	-0.232	0.2714	0.729	1	0.393
Frequency of situations in which clients question vet's advice (1=Never) *(ref.cat.=Frequently/Always)*	0.454	0.6257	0.527	1	0.393
Frequency of situations in which clients question vet's advice (2=Occasionally) *(ref.cat.=Frequently/Always*	0.951	0.3609	6.940	1	0.008
Employment type *(ref. cat.: employed)*	-0.443	0.3338	1.759	1	0.185
age	0.036	0.0102	12.210	1	<0.001
Impact of internet resources on clients' desire to pursue therapy	0.166	0.1073	2.402	1	0.121
Business type *(ref. cat.: corporate-owned)*	-0.206	0.2443	0.709	1	0.400
**Model 2 AUSTRIA: Clients' use of internet resources results in greater expectations of advanced diagnostics and treatments.** ($\chi^2(8)$=5.573, P=0.695)					
Gender *(ref. cat.: female)*	1.091	0.6635	2.706	.	0.100
Percentage of clients using internet resources (1=None-19%) *(ref.cat.: over 60%)*	-0.530	0.7962	0.442	1	0.506
Percentage of clients using internet resources (2=20-59%) *(ref.cat.: over 60%)*	-0.089	0.5044	0.031	1	0.859
Frequency of situations in which clients question vet's	-0.338	1.4315	0.056	1	0.813

advice (1=Never) *(ref.cat.=Frequently/Always)*					
Frequency of situations in which clients question vet's advice (2=Occasionally) *(ref.cat.=Frequently/Always*	-0.100	1.3919	0.005	1	0.943
Employment type *(ref. cat.: employed)*	-0.120	0.7155	0.028	1	0.867
age	-0.012	0.0345	0.117	1	0.732
Impact of internet resources on clients' desire to pursue therapy	0.315	0.2009	2.455	1	0.117
**Model 2 DENMARK: Clients' use of internet resources results in greater expectations of advanced diagnostics and treatments.** ($\chi^2(9)=17.115$, P=0.047)					
Gender *(ref. cat.: female)*	0.752	0.4757	2.324	1	0.127
Percentage of clients using internet resources (1=None-19%) *(ref.cat.: over 60%)*	-0.485	0.5183	0.875	1	0.350
Percentage of clients using internet resources (2=20-59%) *(ref.cat.: over 60%)*	-0.121	0.3970	0.092	1	0.761
Frequency of situations in which clients question vet's advice (1=Never) *(ref.cat.=Frequently/Always)*	0.192	0.7786	0.061	1	0.805
Frequency of situations in which clients question vet's advice (2=Occasionally) *(ref.cat.=Frequently/Always*	-0.140	0.4432	0.100	1	0.751

Employment type *(ref. cat.: employed)*	-0.442	0.4269	1.073	1	0.300
age	0.039	0.0181	4.684	1	0.030
Impact of internet resources on clients' desire to pursue therapy	0.242	0.1442	2.814	1	0.093
Business type *(ref. cat.: corporate-owned)*	0.750	0.4754	2.487	1	0.115
**Model 2 UK: Clients' use of internet resources results in greater expectations of advanced diagnostics and treatments.** ($\chi^2(9)=59.542$, P<0.001)					
Gender *(ref. cat.: female)*	-0.196	0.2844	0.477	1	0.490
Percentage of clients using internet resources (1=None-19%) *(ref.cat.: over 60%)*	-0.192	0.4319	0.197	1	0.657
Percentage of clients using internet resources (2=20-59%) *(ref.cat.: over 60%)*	-0.020	0.2759	0.005	1	0.942
Frequency of situations in which clients question vet's advice (1=Never) *(ref.cat.=Frequently/Always)*	-2.344	0.6516	12.945	1	<0.001
Frequency of situations in which clients question vet's advice (2=Occasionally) *(ref.cat.=Frequently/Always*	-1.261	0.3562	12.525	1	<0.001
Employment type *(ref. cat.: employed)*	0.331	0.3277	1.018	1	0.313
age	0.025	0.0102	5.870	1	0.015

Impact of internet resources on clients' desire to pursue therapy	0.524	0.1138	21.159	1	<0.001
Business type *(ref. cat.: corporate-owned)*	-0.503	0.2486	4.090	1	0.043
**Model 3 AUSTRIA: Clients' use of internet resources improves the discussion about diagnostic and treatment options as the clients have greater knowledge.** ($\chi^2(8)=7.986$, P=0.435)					
Gender *(ref. cat.: female)*	0.238	0.6481	0.190	1	0.663
Percentage of clients using internet resources (1=None-19%) *(ref.cat.: over 60%)*	0.440	0.7391	0.354	1	0.552
Percentage of clients using internet resources (2=20-59%) *(ref.cat.: over 60%)*	0.307	0.5051	0.369	1	0.543
Frequency of situations in which clients question vet's advice (1=Never) *(ref.cat.=Frequently/Always)*	2.799	1.3545	4.271	1	0.039
Frequency of situations in which clients question vet's advice (2=Occasionally) *(ref.cat.=Frequently/Always*	1.713	1.3142	1.699	1	0.192
Employment type *(ref. cat.: employed)*	0.756	0.6655	-0.548	1	0.256
age	-0.011	0.0326	0.113	1	0.737
Impact of internet resources on clients' desire to pursue therapy	0.003	0.1913	0.000	1	0.988

**Model 3 DENMARK: Clients' use of internet resources improves the discussion about diagnostic and treatment options as the clients have greater knowledge.** ($\chi^2(9)$=9.300, P=0.410)					
Gender *(ref. cat.: female)*	0.840	0.5121	2.692	1	0.101
Percentage of clients using internet resources (1=None-19%) *(ref.cat.: over 60%)*	-0.480	0.5243	0.838	1	0.360
Percentage of clients using internet resources (2=20-59%) *(ref.cat.: over 60%)*	-0.169	0.3976	0.181	1	0.670
Frequency of situations in which clients question vet's advice (1=Never) *(ref.cat.=Frequently/Always)*	1.325	0.7807	2.882	1	0.090
Frequency of situations in which clients question vet's advice (2=Occasionally) *(ref.cat.=Frequently/Always*	0.733	0.4537	2.612	1	0.106
Employment type *(ref. cat.: employed)*	-0.139	0.4277	0.105	1	0.746
age	0.021	0.0193	1.139	1	0.286
Impact of internet resources on clients' desire to pursue therapy	-0.058	0.1512	0.147	1	0.208
Business type *(ref. cat.: corporate-owned)*	0.625	0.4960	1.587	1	0.208
**Model 3 UK: Clients' use of internet resources improves the discussion about diagnostic and treatment options as the clients have greater knowledge.** ($\chi^2(9)$=23.512, P=0.005)					
Gender *(ref. cat.: female)*	-0.594	0.2665	4.973	1	0.026

Percentage of clients using internet resources (1=None-19%) *(ref.cat.: over 60%)*	-0.575	0.4289	1.798	1	0.180
Percentage of clients using internet resources (2=20-59%) *(ref.cat.: over 60%)*	0.126	0.2632	0.228	1	0.633
Frequency of situations in which clients question vet's advice (1=Never) *(ref.cat.=Frequently/Always)*	-0.064	0.6167	0.011	1	0.917
Frequency of situations in which clients question vet's advice (2=Occasionally) *(ref.cat.=Frequently/Always*	0.198	0.3404	0.338	1	0.561
Employment type *(ref. cat.: employed)*	-0.373	0.3173	1.379	1	0.240
age	0.041	0.0100	16.835	1	<0.001
Impact of internet resources on clients' desire to pursue therapy	0.071	0.1029	0.475	1	0.491
Business type *(ref. cat.: corporate-owned)*	0.171	0.2375	0.520	1	0.471
**Model 4 AUSTRIA: Clients' use of internet resources can lead to situations where clients are better informed than I am.** ($\chi^2(8)$=13.724, P=0.089)					
Gender *(ref. cat.: female)*	-0.953	0.7322	1.695	1	0.193
Percentage of clients using internet resources (1=None-19%) *(ref.cat.: over 60%)*	-0.083	0.7380	0.013	1	0.910

Percentage of clients using internet resources (2=20-59%) *(ref.cat.: over 60%)*	0.929	0.5179	3.215	1	0.073
Frequency of situations in which clients question vet's advice (1=Never) *(ref.cat.=Frequently/Always)*	-4.033	1.4915	7.310	1	0.007
Frequency of situations in which clients question vet's advice (2=Occasionally) *(ref.cat.=Frequently/Always*	-3.757	1.4813	6.432	1	0.011
Employment type *(ref. cat.: employed)*	-0.215	0.6511	0.109	1	0.741
age	-0.043	0.0331	1.706	1	0.192
Impact of internet resources on clients' desire to pursue therapy	0.127	0.2005	0.400	1	0.527
**Model 4 DENMARK: Clients' use of internet resources can lead to situations where clients are better informed than I am.** ($\chi^2(9)$=11.776, P=0.226)					
Gender *(ref. cat.: female)*	-0.766	0.4745	2.608	1	0.106
Percentage of clients using internet resources (1=None-19%) *(ref.cat.: over 60%)*	-0.164	0.4942	0.111	1	0.739
Percentage of clients using internet resources (2=20-59%) *(ref.cat.: over 60%)*	-0.2471	0.3926	0.395	1	0.530
Frequency of situations in which clients question vet's advice (1=Never) *(ref.cat.=Frequently/Always)*	-0.625	0.7773	0.646	1	0.422

Frequency of situations in which clients question vet's advice (2=Occasionally) *(ref.cat.=Frequently/Always*	0.440	0.4548	0.934	1	0.334
Employment type *(ref. cat.: employed)*	-0.786	0-4231	3.453	1	0.063
age	0.038	0.0177	4.575	1	0.032
Impact of internet resources on clients' desire to pursue therapy	-0.109	0.1317	0.690	1	0.406
Business type *(ref. cat.: corporate-owned)*	0.258	0.5005	0.265	1	0.606
**Model 4 UK: Clients' use of internet resources can lead to situations where clients are better informed than I am.** (χ^2(9)=16.414, P=0.059)					
Gender *(ref. cat.: female)*	-0.713	0.2798	6.943	1	0.008
Percentage of clients using internet resources (1=None-19%) *(ref.cat.: over 60%)*	-0.500	0.4098	1.488	1	0.223
Percentage of clients using internet resources (2=20-59%) *(ref.cat.: over 60%)*	0.226	0.2604	0.753	1	0.385
Frequency of situations in which clients question vet's advice (1=Never) *(ref.cat.=Frequently/Always)*	-0.011	0.6117	0.000	1	0.985
Frequency of situations in which clients question vet's advice (2=Occasionally) *(ref.cat.=Frequently/Always*	0.414	0.3414	1.468	1	0.226

Employment type *(ref. cat.: employed)*	-0.017	0.3181	0.003	1	0.957
age	0.020	0.0096	4.421	1	0.035
Impact of internet resources on clients' desire to pursue therapy	0.064	0.1030	0.390	1	0.533
Business type *(ref. cat.: corporate-owned)*	-0.323	0.2331	1.916	1	0.166
**Model 5 AUSTRIA: Clients' use of internet resources causes clients to form strong opinions so that veterinarians have to justify their diagnostic and therapeutic steps.** ($\chi^2(8)=27.213$, $P<0.001$)					
Gender *(ref. cat.: female)*	0.272	0.6399	0.181	1	0.033
Percentage of clients using internet resources (1=None-19%) *(ref.cat.: over 60%)*	-1.646	0.7709	4.561	1	0.033
Percentage of clients using internet resources (2=20-59%) *(ref.cat.: over 60%)*	-1.530	0.5633	7.376	1	0.007
Frequency of situations in which clients question vet's advice (1=Never) *(ref.cat.=Frequently/Always)*	-3.549	1.5486	5.5251	1	0.022
Frequency of situations in which clients question vet's advice (2=Occasionally) *(ref.cat.=Frequently/Always*	-1.568	1.5283	1.053	1	0.305
Employment type *(ref. cat.: employed)*	1.259	0.7169	3.085	1	0.079
age	-0.067	0.0337	4.003	1	0.045

Impact of internet resources on clients' desire to pursue therapy	0.002	0.2043	0.000	1	0.993
**Model 5 DENMARK: Clients' use of internet resources causes clients to form strong opinions so that veterinarians have to justify their diagnostic and therapeutic steps.** ($\chi^2(9)=44.178$, $P<0.001$)					
Gender *(ref. cat.: female)*	0.551	0.5146	1.145	1	0.258
Percentage of clients using internet resources (1=None-19%) *(ref.cat.: over 60%)*	-0.626	0.5460	1.314	1	0.252
Percentage of clients using internet resources (2=20-59%) *(ref.cat.: over 60%)*	-0.327	0.4254	0.592	1	0.442
Frequency of situations in which clients question vet's advice (1=Never) *(ref.cat.=Frequently/Always)*	-2.941	0.7917	13.798	1	<0.001
Frequency of situations in which clients question vet's advice (2=Occasionally) *(ref.cat.=Frequently/Always*	-1.064	0.4759	4.996	1	0.025
Employment type *(ref. cat.: employed)*	-0.588	0.4394	1.793	1	0.181
age	-0.045	0.0188	5.711	1	0.017
Impact of internet resources on clients' desire to pursue therapy	0.496	0.1460	11.551	1	<0.001
Business type *(ref. cat.: corporate-owned)*	0.912	0.5326	2.943	1	0.087

**Model 5 UK: Clients' use of internet resources causes clients to form strong opinions so that veterinarians have to justify their diagnostic and therapeutic steps.** ($\chi^2(9)=49.313$, $P<0.001$)					
Gender *(ref. cat.: female)*	-0.161	0.2772	0.336	1	0.562
Percentage of clients using internet resources (1=None-19%) *(ref.cat.: over 60%)*	-0.616	0.4140	2.216	1	0.137
Percentage of clients using internet resources (2=20-59%) *(ref.cat.: over 60%)*	-0.222	0.2644	0.705	1	0.401
Frequency of situations in which clients question vet's advice (1=Never) *(ref.cat.=Frequently/Always)*	-2.325	0.6240	13.886	1	<0.001
Frequency of situations in which clients question vet's advice (2=Occasionally) *(ref.cat.=Frequently/Always*	-0.961	0.3349	8.228	1	0.004
Employment type *(ref. cat.: employed)*	-0.030	0.3326	0.008	1	0.929
age	-0.028	0.0101	7.828	1	0.005
Impact of internet resources on clients' desire to pursue therapy	0.218	0.1062	4.216	1	0.040
Business type *(ref. cat.: corporate-owned)*	-0.369	0.2430	2.305	1	0.129
**Model 6 AUSTRIA: Clients' use of internet resources creates a wrong impression of modern small animal practice.** ($\chi^2(8)=15.773$, $P=0.046$)					
Gender *(ref. cat.: female)*	-1.637	0.6647	6.068	1	0.014

Percentage of clients using internet resources (1=None-19%) *(ref.cat.: over 60%)*	-0.262	0.7645	0.117	1	0.732
Percentage of clients using internet resources (2=20-59%) *(ref.cat.: over 60%)*	0.604	0.5513	-1.199	1	0.273
Frequency of situations in which clients question vet's advice (1=Never) *(ref.cat.=Frequently/Always)*	-3.272	1.2546	6.803	1	0.009
Frequency of situations in which clients question vet's advice (2=Occasionally) *(ref.cat.=Frequently/Always*	-2.148	1.1984	3.212	1	0.073
Employment type *(ref. cat.: employed)*	0.348	0.6723	0.268	1	0.605
age	0.008	0.0310	0.063	1	0.802
Impact of internet resources on clients' desire to pursue therapy	-0.188	0.275	0.750	1	0.387
**Model 6 DENMARK: Clients' use of internet resources creates a wrong impression of modern small animal practice.** ($\chi^2(9)$=16.256, P=0.062)					
Gender *(ref. cat.: female)*	0.298	0.5106	0.342	1	0.559
Percentage of clients using internet resources (1=None-19%) *(ref.cat.: over 60%)*	-0.867	0.5298	2.679	1	0.102
Percentage of clients using internet resources (2=20-59%) *(ref.cat.: over 60%)*	-0.301	0.4158	0.524	1	0.469

Frequency of situations in which clients question vet's advice (1=Never) *(ref.cat.=Frequently/Always)*	-1.668	0.7432	5.038	1	0.025
Frequency of situations in which clients question vet's advice (2=Occasionally) *(ref.cat.=Frequently/Always*	-0.336	0.4703	0.511	1	0.475
Employment type *(ref. cat.: employed)*	-0.225	0.4438	0.256	1	0.613
age	-0.020	0.0184	1.195	1	0.274
Impact of internet resources on clients' desire to pursue therapy	0.164	0.1492	1.201	1	0.273
Business type *(ref. cat.: corporate-owned)*	0.618	0.5507	1.261	1	0.261
**Model 6 UK: Clients' use of internet resources creates a wrong impression of modern small animal practice.** (χ^2(9)=40.031, P<0.001)					
Gender *(ref. cat.: female)*	0.047	0.2717	0.030	1	0.863
Percentage of clients using internet resources (1=None-19%) *(ref.cat.: over 60%)*	-0.764	0.4294	3.170	1	0.075
Percentage of clients using internet resources (2=20-59%) *(ref.cat.: over 60%)*	-0.281	0.2683	1.137	1	0.286
Frequency of situations in which clients question vet's advice (1=Never) *(ref.cat.=Frequently/Always)*	-2.624	0.6240	17.682	1	<0.001

Frequency of situations in which clients question vet's advice (2=Occasionally) *(ref.cat.=Frequently/Always*	-0.668	0.339	4.001	1	0.045
Employment type *(ref. cat.: employed)*	0.160	0.3253	0.242	1	0.623
age	-0.004	0.0099	0.174	1	0.677
Impact of internet resources on clients' desire to pursue therapy	0.265	0.1073	6.080	1	0.014
Business type *(ref. cat.: corporate-owned)*	-0.261	0.2414	1.166	1	0.280
